# Abstracts and Gleanings

**Published:** 1887-03

**Authors:** 


### ABSTRACTS AND GLEANINGS.

**Health of Los Angeles, California, and Vicinity.**—Dr. W. Lindley in an address before the Society of the County of Kings:

DISEASES OF CHILDREN.

There summer disturbances of the intestinal tract are never prevalent. I have been physician to the Los Angeles Orphans' Home for the last six years, and, although there are about eighty inmates, many of them under two years of age, yet in all that time there has not been a death from the so-called cholera infantum or any disease of similar nature. Scarlet fever, diphtheria, measles, and whooping cough usually run a very mild course. During the six years there have been two epidemics of scarlet fever in the home and but two deaths, and no deaths from the other diseases mentioned. I have never known the child, born in Southern California, of a phthisical parent to die of pulmonary disease.

DYSPEPSIA.

Dyspeptic persons are almost invariably relieved, owing, in part, to the character of food always available. Every variety of fresh vegetables can be had all the year; strawberries are always in the market; oranges, lemons and limes are in season during winter and spring; apricots, nectarines, peaches, and several varieties of berries, ripen during May, June and July, and apples and grapes are in season until the orange again ripens. California flour is unexcelled. Beef, mutton, fish, the products of the dairy, and many varieties of game, complete a range of diet that is within reach of all.

MALARIAL DISEASES.

Intermittent fever never develops in Los Angeles county. I speak thus positively because my experience as Health Officer of the city of Los Angeles, Attending Physician to the Los Angeles County Hospital of 100 beds, as well as my private practice, justifies me in the statement. I believe the same can be said of the other counties indicated as the health region of Southern California. On the other hand, I have had numerous patients with ague that have come saturated with malaria from certain portions of the San Joaquin Valley. A short residence in Los Angeles, assisted by ordinary treatment, cures them, and a permanent residence gives them perfect immunity.

HAY FEVER.

A few months since I became interested in this subject, and, after corresponding with fifty of the leading physicians in the counties mentioned, arrived at the following conclusions:

"1. Hay fever never originates in Southern California.

"2. All persons with hay fever that have come, seeking relief, to Southern California, have been benefited; almost all have been cured.

"3. That a few miles inland, in the foothills, relieves such as are not benefited by a residence at the seaside."

### ASTHMA.

The great majority of persons afflicted with this disease can get permanent relief within a radius of sixty miles of Los Angeles; many have perfect immunity in the city or its immediate vicinity, but Riverside, sixty miles east, and Newhall and Ravenna, thirty miles north, are the most noted resorts for asthmatics, although I have had some patients that could live only on the sea-coast. Among these is my friend Dr. E. C. Folsom (Harvard,) who, after vainly trying many famous resorts in Europe and America, found perfect health at Santa Monica, where he is doing an extensive practice.

### SENILITY.

This is a paradise for persons who have passed the meridian of life. Instead of spending the most of their lives in rooms artificially heated, they get a new lease of life from the sun's rays, the pure atmosphere, and inspiring surroundings.

### NERVOUS PROSTRATION.

Persons in this condition receive benefit from the pleasure of out-door life and the refreshing sleep that comes at night. No matter how warm the day, the nights are invariably cool enough to encourage rest. There is something soothing in the very atmosphere.

### CHRONIC RHEUMATISM.

Besides the beneficial effects of the climate on this disease, there are a great variety of mineral springs, hot and cold, that have quite a local reputation, and about which an interesting volume might be written.

### PHTHISIS.

In an interesting paper on "The Climatic Treatment of Phthisis," Dr. Harold J. Williams says: "An ideal health resort for this disease should be sparsely and newly settled. It should possess a pure water supply and adequate drainage. It should be of a dry and porous soil, and should be favorably situated with respect to neighboring heights and marshes and prevailing winds. It should be equable in temperature, and should possess the maximum of pleasant weather. It should not be so hot as to be enervating, nor so cold as to prevent out-door exercises and proper ventilation of houses. It should afford plenty of amusement; it should not be crowded with consumptives, and it should be sufficiently unfashionable to admit of hygienic dress.

Dr. Tyndale, in his work on the treatment of consumption, says dryness is the chief factor of an ideal climate, and a certain equability of temperature the next.

Dr. Williams, of London, says in the majority of cases of chronic phthisis, and especially in women whose circulation is weaker, the warm and dry climates are the best.

Parks, in his work on "Practical Hygiene," says: "The best climates for phthisis are those which permit the greatest number of hours to be passed out of the house."

Professor Loomis, in his work on "Diseases of the Respiratory Tract," says: "Endeavor to select a climate where he may be out of doors every day and at any hour of the day."

Within a randius of ten miles of Los Angeles all the requirements mentioned by the distinguished authorities quoted above can be found in numerous points happily combined.

**Arsenic and Antipyrine in Fever.**—I shall here relate the clinical history of a few cases in which I combined these two remedies, antipyrine and arsenic, omitting, however, minor symptoms and complications, and the measures used to meet them:

CASE I.

*Intermittent Fever, Quotidian Type.*—Adult, male, had chill at 8 a.m., July 10, second paroxysm followed by moderately high fever. Saw patiept at 12 m.; temperature 101.5° F.; fever had already begun to decline. The patient, quite an intelligent man, refused to take quinine or any derivative of cinchona, because it produced an eruption, which he described as "terrific" and far worse than ague. More to test its effect than because he needed it, I gave 15 grains of antipyrine and directed that he take 5 drops of Fowler's solution every three hours for the next four days, and if the paroxysms were arrested then to continue it t. i. d. for several days longer, cautioning the patient to omit for twenty-four hours if there should develop much swelling of the eyelids. Also left a 15-grain dose of antipyrine to be given next day if there should be much fever. Patient reported a few days later that the antipyrine acted promptly in reducing fever. There was a slight chill next day, with a rise of fever, which was promptly checked by the antipyrine. After this, no more symptoms of the ague. No unpleasant effects from the arsenic.

CASE II.

*Remittent Fever.*—Adult, female; seen August 14; temperature 102.4° F. Previous history of a remitting fever of three or four days duration. She had been taking from 4 to 6 grains of quinine per day. She objected strongly to taking quinine in increased doses, from the cerebral excitement which it produced, or as she expressed it, "it made her crazy." I prescribed, however, 25 grains in divided doses in twenty-four hours. Next day I was hastily summoned, and found her madly delirious, with a temperature only 101.5°. Morphine to quiet delirium, 10 grains of antipyrine to reduce temperature—one dose per day—and 5 drops of Fowler's solution every four hours, from 6 a. m. to 10 p m. Fever disappeared in four days, but convalescence was not complete for two weeks.

CASE III.

*Remittent Fever, Bilious Type.*—Adult, female; seen August 20. This patient had fever for three days with a morning temperature of from 102° to 102.5°; evening from 103° to 104.8°. Took

30 grains of quinine a day. At 4 p. m., evening of the third day, temperature 104.8°. Had given 40 grains of quinine this day with not the slightest decline of fever. Conjunctiva injected ; some delirium. Gave 15 grains of antipyrine ; at 5 p. m. temperature 103°; gave 15 grains more of antipyrine. Shortly after this profuse diaphoresis was set up, and at 9 p. m. temperature was 98.5° ; stomach had been very irritable for three days, but patient now took some nourishment and passed a quiet night. Gave no more quinine or antipyrine, as there was no return of fever. Patient was up in her room in a few days, but in just one week there was a relapse. I then gave from 15 to 20 grains of quinine every forenoon, and one dose of 15 grains of antipyrine every afternoon. Temperature varied from 100° to 101.5° a. m. to 102° to 103° p. m. Antipyrine invariably caused a reduction of from two to three degrees, and there would not be a subsequent rise until the next morning. This attack lasted one week, and as the patient cou d not be induced to take quinine when there was no fever, was followed by another relapse. The same treatment was followed as in the last attack, with the addition of 5 drops of Fowler's solution t. i. d. This was kept up until convalescence was complete.

### CASE IV.

*Malarial Fever.*—Adult, female ; temperature varied from 100° to 103°, with irregular exacerbations. Seen first July 19. Twenty-five grains of quinine were given per diem for one week, until temperature did not exceed 100° at any time during the day. There was great nervous excitement. Patient had not slept for several days and nights, with the exception of short naps induced by ⅛-grain doses of morphia. The bromides and chloral had no effect whatever. I then gave 5 drops of Fowler's solution every four hours, and directed that 15 grains of antipyrine be given whenever the temperature went to 101°. Nervous symptoms abated, appetite improved, and there was some return of strength. Temperature did not decline further, but on the fourth day of the treatment began to rise, and on the evening of the sixth went up to 102.5°. Went back to quinine, giving 30 grains from 6 a. m. to 12 m., and from 20 to 30 grains of antipyrine every afternoon. Fever showed no disposition to yield at all, and quinine was gradually increased until 60 grains per diem were reached. Patient complained of very little deafness until this dose was reached. This was the eighteenth day of the treatment, and the twenty-first of her sickness. This dose was given for two days, with the effect of bringing the highest temperature, marking down to 101.5°. Antipyrine was discontinued on the sixteenth day. It always produced a profuse perspiration, and seemed to depress the heart so much that I was afraid to use it any longer. After exhibiting 60 grains of quinine for two days, the dose was reduced 5 grains a day until 25 grains were reached. After the middle of the fourth week temperature never went above 100°, but did not remain at the normal until the end of the sixth week. When quinine was resumed in this case, it did not produce the nervous excitement that it caused at first, even when 1 drachm daily was given.

In Case I, it might be objected that it was quite a mild attack, and the arsenic would have done just as well without the antipyrine. Probably it would, but if ever I have to treat a severe attack of intermittent in this patient, I shall have great confidence that I can keep his temperature under control with antipyrine until the arsenic shall have eradicated the malaria.

In Case III, the action of the antipyrine, in bringing the temperature to normal so promptly, was wonderful. Can we explain it by supposing that the quinine administered had already destroyed the malarial poison, but that the heat centre in the medulla remained so perturbed that the temperature did not decline until there was direct action upon this centre by the antipyrine? This may have been a coincidence, but the result was certainly most gratifying. I was strongly impressed that this case was about to assume a pernicious character, from the condition of the conjunctivæ and the delirium.

Case IV is reported because, in this instance, the combined action of antipyrine and arsenic was not gratifying. The nervous symptoms quickly disappeared, although the fever did not subside, but rose again in a very intense form. Hence, it is important to recognize that this is not a specific treatment. But from these cases and a number of others in which I have used antipyrine and arsenic—when quinine either could not be used at all, or when it was best to suspend its use for a few days—I am convinced that the combined use of these two drugs meets a want that has long been felt by those who have much malaria to treat.

I have never seen any rash caused by antipyrine. Occasionally its administration is followed by nausea. I usually exhibit it in solution in ice-cold water. Given in this way the bitter taste is very transient. If ice-water is not convent it is best to give the drug in capsules. * * *—Dr. D. B. FRONTIS, of Wadesborough, N. C., in *Medical News*

**Bromide of Ethyl in Conjunction with Chloroform.**—Dr. J. J. Chisolm is now employing, at Baltimore Academy of Medicine, the bromide of ethyl in conjunction with chloroform as an anæsthetic. His method is to anæsthetize the patient first with bromide of ethyl, and then, after discontinuing this agent, to keep up the anæsthesia with chloroform. The advantage claimed by him for this method is that he accomplished the desired result much more quickly than when chloroform is used alone.

Dr. H. P. C. Wilson has been very forcibly impressed with the universal absence of nausea in all patients whom he has seen chloroformed abroad. He has had opportunity to see a large number of persons anæsthetized in Europe, and has never yet observed nausea in any of them. He has made enquiry, but can get no satisfactory explanation for its absence.

Dr. C. C. Bombaugh asked Dr. Wilson if the frequency of nausea in his cases might not be attributable to impurities in the chloroform. He said ofttimes aldehyde is present in chloroform as an impurity. In his experience in army surgery he had used it freely and had no cases of nausea.

Dr. H. P. C. Wilson replied that he had no reason to suspect the drug used by him, as he never employed any other than Squibb's.

Dr. Samuel Theobald has avoided nausea to a great extent by giving a hypodermic injection of morphia and atropia about thirty minutes before the time for administering the chloroform. The period of recovery after this treatment is quiet and without excitement.

Dr. C. C. Bombaugh asked Dr. Chisolm if he had noticed any decrease in the number of patients nauseated since he has used bromide of ethyl in the early stages of the process of anæsthesia.

Dr. J. J. Chisolm said he had not seen much nausea lately, but could not say that the decrease was due to his plan of operating.

Dr. Chistopher Johnston said it was singular how a patient would at one time take an anæsthetic with but little or no discomfort, and at another time the result would be almost fatal. He referred to a child with harelip upon whom he had occasion to operate. The fissure was of such a size as to necessitate two operations. The first time he brought the child under the influence of the anæsthetic there was nothing to create alarm, but at the second operation the patient became almost lifeless.

### TRACHEOTOMY FOR MEMBRANOUS CROUP.

Dr. Christopher Johnston spoke of an interesting tracheotomy that he had performed. The history is as follows: On the night of October 6th he was called to see a child, of about three years of age, suffering from membranous croup. The patient was then very ill, stridulous breathing was present. On the morning of October 7th he did tracheotomy, using a Durham tube. For a time the operation gave much relief. For a period of five to seven days there was no attempt to breathe through the larynx, but all the respiration was carried on through the tube in the trachea. After this time there was a considerable swelling of the tissues of the neck, the membranous formation could be seen in the trachea below the point of insertion of the tube. The child's strength was supported by the constant administration of brandy. The upper part of the trachea and larynx was completely occluded by false membranes, which were dislodged by passing upward from the opening in the trachea a director to which was attached a piece of string tied to a bit of linen cloth. Intubation was also resorted to. Paralysis of the throat muscles allowed food to pass into the trachea, and it was extended through the tracheal tube. No air passed through the larynx for nine days. The tube was in the child's neck, in all, a little more than three weeks. After the removal of the tube the child grewbetter, and is now perfectly well. He calls attention to this case as being somewhat an exception to the rule, for it is very rare that a tube can be removed from the trachea at all after it has remained there so long as this one did.

Dr. J. Edwin Michael thinks there are many children whose lives are sacrificed by the neglect of this operation.

Dr. Christopher Johnston thinks one justified in doing this operation for no other purpose than to relieve suffocation. He had also another child suffering from membranous croup or diphtheria, in whom he used a spray of nitrate of silver solution. The tissues were supplied with nutriment by obliging the child to breathe an atmosphere of pure oxygen. It recovered from this attack, but eighteen months later he was called on to do tracheotomy on the same child who had contracted the disease again. The child recovered, but the tube could never be removed. A year afterward the child was suffocated by the accidental occlusion of the tube. Autopsy revealed a cicatricial contraction of the tissues of the larynx. Intubation of the larynx was frequently resorted to with no good result.

Dr. J. J. Chisolm saw a patient upon whom tracheotomy had been performed one year ago for syphilitic contraction of the larynx. The tube has remained in position for twelve months, and cannot be removed.

Dr. J. Edwin Michael saw a patient who had stenosis of the larynx. Dilatation had constantly been carried on in order that the man might breathe, but upon one occasion this precaution was neglected and the man began to find beathing becoming more and more difficult until he became quite cyanotic. Dr. H. C. Sherry, who was the physician in attendance, called Dr. Michael in to do tracheotomy. He tried to do laryngotomy, but found that the cricoid cartilage ossified. He then made the opening further down. Relief was immediate. This patient wore the tube for months. Dr. McSherry dilated the larynx from the wound upward until the opening in the larynx was sufficiently large to permit closure of the wound in the trachea. There was some interference with his voice, but the man is now living and in good health.

Dr. Samuel Theobald wished to know if the introduction of these dilators did not give rise to considerable spasm.

Dr. Michael said it did at first, but the muscles soon became tolerant of their presence.

FORWARD DISPLACEMENT OF THE EYE-BALL, DUE TO A TUMOR.

Dr. J. J. Chisolm related a case that was of interest to himself. It was the displacement forward of the eye-ball, due to the presence of a tumor in the socket. The patient wanted the tumor removed if the eye could be returned to its position.

Upon anæsthetizing the patient he found the tumor to be an osteoma of the socket tissues, having its origin in the malar bone. The patient was allowed to come from under the influence of the chloroform and was told of his condition. He concluded not to be operated upon. This is the second of these cases that he has seen in the past year. In his second case the ball was so far pushed out that he removed it because of the disfigurement it gave rise to.

Dr. Christopher Johnston had had a patient with a tumor of the lachrymal gland. It became so large as to push the eye completely into the inner angle of the socket. On removal of the enlarged gland the eye receded to its normal position.

Dr. J. J. Chisolm: Within a week of the patient just referred to by him, saw a patient with the eye bulged out from an enlarged lachrymal gland. He asked Dr. Theobald if he had ever used hydrogen peroxide for clearing the secretions from offensive ears?

Dr. Theobald had not.

Dr. J. J. Chisolm finds it a very nice method. He first washes out the ear with warm water, then pours in the hydrogen peroxide. It causes an oxidation of the secretions, and in a few minutes the ear is perfectly clean.—*N. C. Med. Journal.*

**Ice-Water Enemata in Treatment of Diarrhœa.**—Dr. R. M. Simon, writing in the *British Medical Journal* for October 30, 1886, states that this mode of treatment has frequently been adopted in cases of collapse occuring during diarrhœa in young children at the Birmingham General Hospital. He recommends that ice should be dissolved in water and from 2 to 3 ounces injected. In his experience, which he says has been quite extensive, the immediate effect is good in producing sleep, as is important in the collapsed condition. Subsequently the effect upon the diarrhœa is also good, and it will rarely be found necessary to repeat the enema. Dr. Simon thinks that the cold enemata act by an astringent effect on the loaded vessels of the intestines, and so diminish the intestinal flux. It has sometimes been found to be expedient to give a few drops of brandy about the time of the injection, and perhaps internal medication should be continued. In his experience no depression or bad effect has ever resulted.—*Therapeutic Gazette.*

**Localization of Brain Tumors.**—It is indeed a triumph of science which enables the presence of a tumor in the substance of the brain to be not only diagnosed, but its exact location so clearly determined by the symptoms, that the surgeon confidently operates to remove it, and finds his confidence rewarded by the restoration of the patient to health.

The English medical journals report the fourth case of the successful excision of a tumor in the brain.

The patient was a man who had suffered terrible pain in the head, with occasional convulsions, and had been absolutely paralyzed on one side of his body for a month, and had passed into a semi-comatose condition.

Mr. Victor Horsley trephined over the region of the brain indicated—the motor region of the right hemisphere—and, after enlarging the opening in the skull made by the trephine, removed a tumor from the brain.

The tumor weighed four ounces and a half, and was three inches broad by two inches deep.

On the fourth day after the operation the wound was entirely healed, and the man had recovered some power in the paralyzed leg.

The history of the three other cases in which Mr. Horsely has operated successfully on the *motor area of the cortex of the brain*, was given to the Section of Surgery at the meeting of the Association, and will be shortly published.

It may be interesting to anti-vivisectionists to know that not only have the facts upon which the diagnosis of the seat of the lesion or disease in these cases rests, been discovered by experiments on lower animals, but that the details of treatment have also been worked out in the course of these experiments.—*Ibid*

**Union of Severed Parts.**—A Russian medical journal gives details of two cases occurring in soldiers, one of whom accidentally cut away, with an axe, the second phalanx of his right forefinger; the other, in a similar way, had severed the second phalanx of his left thumb.

In the first case the finger held by a strip of skin, but in the second the chopped-off part was brought away in a glove. In both cases the cut was clean, without any crushing of the parts. The first man was seen by the doctor two hours after the accident, the second three hours.

The wounds werewashed with regulation antiseptic washes, and the severed parts accurately fixed by sutures in their normal situations, after which an iodoform dressing was applied.

In both cases perfect union by the first intention ensued, with return of sensation and limited motion.

These cases confirm the propriety of re-adjusting parts cut away, and maintaining their temperature until union takes place. They should be held on for hours by hand, until the surgeon arrives to secure them by adhesive strips, sutures, bandages, or other means, as required.

If warm water is at hand, a cloth moistened in it, and applied to maintain heat in the severed parts, wi l encourage re-establishment of the circulation.—*Popular Science News.*

**To Prevent Mammary Abscess.**—Although Dr. Goodell ridicules the idea of aborting mammary abscess, which he does not think can be done, yet Mr. Miall (*British Medical Journal*) says that when mammary abscess is on the point of forming, he has frequently seen all the symptoms rapidly disappear in a few hours, under the influence of fomentations with hot water and carbonate of ammonia. He uses an ounce of the carbonate in a pint of water, and when solution is accomplished the temperature of the fluid will be hardly too high for fomentation to be commenced with cloths dipped in the liquid. He applies them for from half an hour to two hours, at the same time protecting the nipples. He has often had immediate relief, and seldom requires to make more than three applications.—*American Medical Digest.*

**Apparent Recovery from Phthisis.**—A patient sent to the Polyclinic in the fall of 1883, supposed to be dying from consumption, is still alive, and apparently healthy, having recovered under treatment by inhalations of compressed air. The diet consisted of raw beef, milk, eggs and green vegetables. The medicine given from time to time, according to indications, were fluid extract of erythoxylon (coca), wine of coca, pill of iodoform and iron, syrup of hypophosphites with iron.—*The Polyclinic.*

**Biniodide of Mercury in Scarlet Fever.**—In the same journal Dr. C. R. Illingworth, alluding to a former communication of his biniodide of mercury in scarlet fever and diphtheria, says: That it is a true specific for the former is proved by the defervescence commencing immediately upon the administration of the medicine, instead of upon the fifth day, and by the absence of desquamation in consequence. That it acts as a specific in the latter is shown by the rapid disappearance of the membranous effusion and reduction of temperature. The efficacy ot the medicine depends, I think, upon the diffusible potassic iodide carrying the germicide biniodide to every portion of the circulation. Prescribed in this form, the biniodide of mercury has not, so far as I am aware, been used before for these diseases.—*New York Med. Journal.*

**Acorn Cocoa in Infantile Diarrhœa.**—Mr. F. W. Elsner, (*Australian Medical Gazette*, *Practioner*) speaks very highly of the efficacy of acorn cocoa in all forms of diarrhœa in children. Acorn cocoa is a preparation of ordinary cocoa powdered and freed from fat, to which are added the soluble parts of roasted acorns without cellulose, a little sugar and roasted flour. A teaspoonful is mixed with cold water, and boiled, being constantly stirred; this may be administered three times a day with a spoon, or placed in the feeding bottle. In twenty-five cases of continuous and exhausting diarrhœa in which he administered this preparation, the benefit was rapid and complete; it never took more than two days to effect an improvement, while twlelve days was the limit at which a complete cure occurred.—*New York Medical Journal.*

**Treatment of Ingrowing Toe-nail.**—Dr. Philip Miall writes to the *British Medical Journal* that he has for many years used tannin for ingrowing nails and not find rest necessary. A concentrated solution (an ounce of perfectly fresh tannic acid dissolved in six drachms of pure water, with a gentle heat) must be painted on the soft parts twice a day. Two cases recently had no pain or lameness after the first application and went about their work immediately, which they could not do before. After about three weeks of this treatment the nail had grown to its proper length and breadth, and the cure was complete. No other treatment of any kind was used, though formerly he introduced lint under the ingrowing edge in such cases.—*Medical and Surgical Reporter.*

**How to Syringe the Ear.**—Dr. H. G. Morse, *Maryland Medical Journal:* "If it be probable that the foreign body is wedged in upon the drum, or, perhaps, pushed through it into the cavity of the tympanum, there is nothing to be done but to remove it all hazards. Perhaps the best way, in case all attempts by means of delicate instruments introduced into the canal (which is at the same time well illuminated by a concave mirror placed on the forehead by means of a band) have failed to reach the body, will be to adopt Troltsch's suggestion and to detach the auricle posteriorly, and thus reach the body from behind. Having thus separated the auricle from its attachment, it will be very easy to remove anything which may rest upon the membrana tympani.—*Medical Digest.*

[I have found no instrument so efficient for removing foreign bodies from the ear, as a very small probe, a little flattened at the end and slightly bent, like a dental scrape, which passing around the foreign body on one side may be made to hook or pull it outward.—ED. SO. MED. RECORD.

**Precaution Against Cholera.**—Surgeon-General Hamilton, of the Marine Hospital Service, returned to Washington last week, from a tour of inspection along the Atlantic and Gulf coast, with a view of ascertaining what safeguards are needed to prevent the introduction of cholera or yellow fever from the south. Dr. Hamilton was accompanied on the tour by the health officers of Charleston, Savannah, Tampa, Sanford, and Jacksonville. The Atlantic coast ports were found to be well protected, but the committee decided that a quarantine hospital is needed near Key West, Fla., and Dr. Hamilton will make a recommendation to that effect. Cuba was also visited, and its entire coast line inspected.—*Medical Reporter.*

**Preventive Inoculation Against Phthisis.**—M. Vittorio Cavagius has reported to the Academie des Sciences, November 29, that a 2 per cent. solution of carbolic acid destroys the infective power of tuberculous virus, that a weaker solution (1.25 per cent.) enfeebles or attenuates it. By inoculation of rabbits and guinea-pigs first with inert tuberculous matter, then with attenuated, he found that they were not made tuberculous, even when pure tuberculous matter was injected.—*Medical Reporter.*

**Ecchinocœa for Typhoid.**—Dr. H. C. F. Myer writes to the *Kansas City Medical Index* that no quinine, whisky, or opium would be required in the treatment of typhoid if the medical profession would give tincture of ecchinocœa angustifolia, or as it is commonly called, black samson root, a trial. The fever will be reduced by it, and it is antiseptic. By its external and internal use it will lower the temperature, will shorten the course of disease, and will save the life of the patient.—*Medical Reporter.*

**A customer** called at a pharmacy in the Rue Grenelle, says a French paper. "Give me something to get rid of worms." "Yes; what kind of worms?" "They are in my wooden leg, and are eating it away."

**Pompous physician** (to patient's wife)—"Why did you delay sending for me until he was out of his mind?"

Wife—"O, doctor, while he was in his right mind he wouldn't let me send for you."

